# What role of the cGAS-STING pathway plays in chronic pain?

**DOI:** 10.3389/fnmol.2022.963206

**Published:** 2022-08-01

**Authors:** Jingxiang Wu, Xin Li, Xiaoxuan Zhang, Wei Wang, Xingji You

**Affiliations:** ^1^Department of Anesthesiology, Shanghai Chest Hospital, Shanghai Jiao Tong University, Shanghai, China; ^2^School of Medicine, Shanghai University, Shanghai, China

**Keywords:** cGAS-STING pathway, chronic pain, inflammation, autophagy, immunity, apoptosis

## Abstract

Chronic pain interferes with daily functioning and is frequently accompanied by depression. Currently, traditional clinic treatments do not produce satisfactory analgesic effects and frequently result in various adverse effects. Pathogen recognition receptors (PRRs) serve as innate cellular sensors of danger signals, sense invading microorganisms, and initiate innate and adaptive immune responses. Among them, cGAS-STING alerts on the presence of both exogenous and endogenous DNA in the cytoplasm, and this pathway has been closely linked to multiple diseases, including auto-inflammation, virus infection, and cancer. An increasing numbers of evidence suggest that cGAS-STING pathway involves in the chronic pain process; however, its role remains controversial. In this narrative review, we summarize the recent findings on the involvement of the cGAS-STING pathway in chronic pain, as well as several possible mechanisms underlying its activation. As a new area of research, this review is unique in considering the cGAS-STING pathway in sensory neurons and glial cells as a part of a broader understanding of pain, including potential mechanisms of inflammation, immunity, apoptosis, and autophagy. It will provide new insight into the treatment of pain in the future.

## Introduction

Chronic pain is classified as neuropathic and inflammatory pain that affects many patients worldwide. In contrast to short-lasting acute pain, chronic pain can be caused by various conditions. Once developed, it may seriously affect the patient's quality of life and cause numerous syndromes. Mechanisms of chronic pain are complex; peripheral nerve lesions or chemical mediators may evoke receptors (nociceptors) sensitive to noxious stimuli in nerve fibers. The spinal cord then processes somatosensory information, further contributing to central sensitization (Descalzi et al., 2015). Typical treatment measures for chronic pain currently include opioid analgesics, non-steroidal anti-inflammatory drugs, and surgical intervention; however, they all have limited long-term benefits and certain risks. For instance, long-term use of opioids in patients will increase the risk of addiction and produce opioid-induced hyperalgesia (Brush, 2012). Meanwhile, surgical intervention may induce postoperative disability, and the recurrence rates are high (Tarnanen et al., 2012). How to alleviate chronic pain and improve patients' prognosis remains a global health problem to be solved. Thus, novel therapeutic targets are urgent and worth developing.

**Figure 1:**
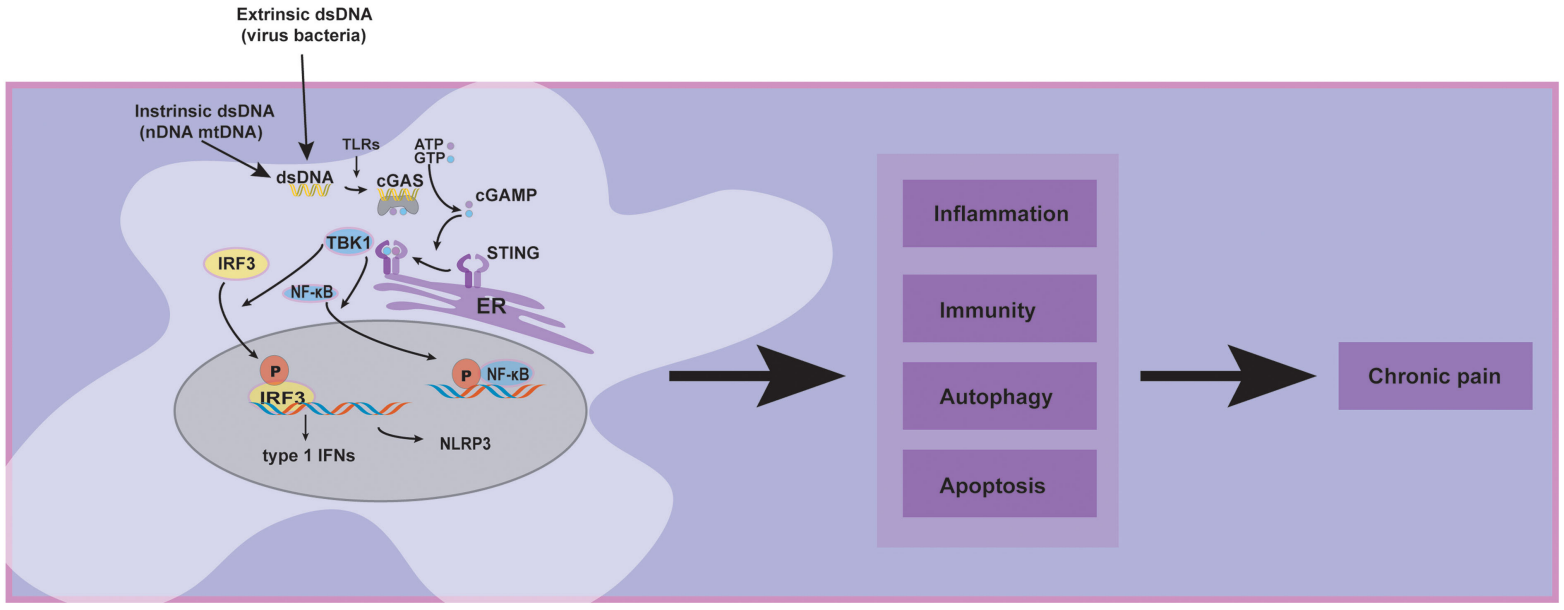
The diversity of cGAS-STING pathway activation in chronic pain and its related pathways.

Recently, special attention has been paid to emphasizing and critically discussing the cGAS-STING pathway, which is a dominant pathway that responds to cytosolic DNA in the context of tumor immunity, cellular senescence, and inflammatory diseases (Ablasser and Chen, 2019). Concerning the roles of the cGAS-STING pathway in chronic pain, it has been reported that activating the cGAS-STING may have various effects. Some studies indicated that STING releases proinflammatory cytokines, which may cause neuroinflammation to aggravate chronic pain (Wang et al., 2019; Tian et al., 2020; Sun et al., 2021). In contrast, another study showed that type 1 interferons over-activated by cGAS-STING pathway might exert an antinociceptive effect in sensory neurons (Donnelly et al., 2021). Today, cGAS-STING pathway in host immunity is more understood, and the therapeutic approaches targeting this pathway show promise for future clinical pain applications. Given the limitations of the current research, we review recent literature focusing on several chronic pain models such as low back pain, bone cancer pain, and spared nerve injury, demonstrating that cGAS-STING pathway is central to the progression and maintenance of chronic pain (Figure 1), but our understanding of its diverse functions on chronic pain still remains incomprehensible.

## Overview of cGAS-STING signaling

Innate immunity is the first line of defense against foreign substances and pathogens, and recognizing DNA is one of the most fundamental aspects of host defense. Pathogen recognition receptors (PRRs) serve as innate cellular sensors of danger signals, detect invading microorganisms, and initiate innate and adaptive immune responses (Thompson et al., 2011). PRRs comprised of toll-like receptors (TLRs), retinoic acid-inducible gene I-like receptors (RLRs), nucleotide oligomerization domain-like receptors (NLRs, also called NACHT, LRR, and PYD domain proteins), and cytosolic DNA sensors (Thompson et al., 2011). In 2008, several research teams discovered a new protein named “stimulator of interferon genes” (STING), which is the major type of innate immune receptor in the endoplasmic reticulum (ER) (Ishikawa and Barber, 2008). Bacteria-derived cyclic di-guanylate monophosphate (c-dGMP) or cyclic di-adenosine monophosphate (c-dAMP) were confirmed to be ligands for STING (Burdette et al., 2011).

First, cGAS is activated upon dsDNA recognition, undergoes an allosteric structural change, and subsequently catalyzes the synthesis of cyclic dinucleotide GMP-AMP (cGAMP) (Wan et al., 2020). Second, cGAMP interacts with and activates STING, which is a multi-structure transmembrane protein mainly located in the endoplasmic reticulum (ER). Subsequently, STING translocates from ER to ER-Golgi intermediate compartment (ERGIC) and afterward to the Golgi. Third, the c-terminal tail of the STING recruits TBK1 through a conserved PLPLRT/SD amino acid-binding motif and promotes the autophosphorylation of TBK1 in Golgi. After TBK1 phosphorylation, the complex of STING and TBK1 may further recruit IRF3 to phosphorylate it (Couillin and Riteau, 2021). IRF3 phosphorylation aimed to promote its dimerization, nuclear translocation, and target gene induction of type 1 interferons. Additionally, IRF3 activity is essential for inducing numerous target genes, including genes encoding inflammasome (such as NLRP3) and proinflammatory cytokines (Li et al., 2019). Another major signaling module in the STING pathway is that TBK1 may promote NF-κB activation by triggering nuclear translocation of NF-κB (Yum et al., 2021).

## The activators of the cGAS-STING pathway in chronic pain

*In vivo*, host detection of pathogen-derived nucleic acids by pathogen recognition receptors (PRRs) is involved in context-dependent recognition of intrinsic dsDNA and extrinsic dsDNA (Thompson et al., 2011). Notably, emerging studies paid attention to PRRs as molecules involved in the pathogenesis of neuropathic pain (Kato et al., 2016). Therefore, it is reasonable to believe that the potential release of extrinsic and intrinsic dsDNA may activate the cGAS-STING pathway in chronic pain.

### Activated by extrinsic DsDNA

Extrinsic dsDNA, from pathogens such as viruses, bacteria, and parasites, can be internalized into the cytosol in several ways to activate the cGAS-STING pathway (Diamond et al., 2018). The patrol nuclease degrades the dsDNA entering the cell in the lysosome or cytoplasm. In cells with abundant dsDNA or a lack of nuclease, internalized DNA may remain in the cytoplasm and be converted into cGAMP to activate STING (Ablasser et al., 2013). Currently, the evidence indicates that SARS-CoV-2, the etiologic agent of COVID-19, can damage and alter the nervous system, causing headaches and myalgia (Abboud et al., 2020). Another report has suggested that the pain may be related to the increase of type 1 interferons, which is the downstream of STING in young patients (Papa et al., 2021). Simultaneously, an animal experiment has also proved that viral infection induces pain by directly affecting nociceptors in dorsal root ganglion with type 1 interferons (Barragán-Iglesias et al., 2020). The above studies suggest that extrinsic dsDNA-like viruses and bacteria may cause pain *via* activating the cGAS-STING pathway.

### The intrinsic self-dsDNA may activate cGAS-STING in chronic pain

Furthermore, extrinsic dsDNA and intrinsic dsDNA can activate cGAS-STING pathway and induce chronic pain. For example, self-dsDNA was increased and mediated the activation of cGAS-STING pathway in the spinal cord, contributing to the spared nerve injury (SNI)-induced neuropathic pain (Sun et al., 2021). The intrinsic self-dsDNA is composed of nDNA and mtDNA, which can be segregated inaccurately and released into the cytosol, triggering the STING pathway (Bhattacharya et al., 2017). Additionally, in the peripheral neuropathy pain model, DNA damage and the nuclear DNA released are associated with pain production (Canta et al., 2015), suggesting that STING may be activated in that pain model. Another intrinsic dsDNA was mtDNA, which is a component of mitochondria that belongs to the only non-nuclear genome. Compared with nDNA, mtDNA is more unstable and vulnerable to the effects of oxidative stress due to its proximity to mitochondrial reactive oxygen species (mtROS) and a lack of repair machinery. mtDNA stress may contribute to the cGAS-STING pathway activation and type 1 interferon responses in various pathological states, including infectious diseases, cancer, neurodegeneration, and other mitochondria-related illnesses (Zhou et al., 2021). Importantly, it has been established that mtDNA depletion inhibited the cGAS-STING pathway and nuclear translocation of p65 and IRF3 (Zhou et al., 2021).

Additionally, mitochondrial dysfunction contributes to the etiology of pain, as indicated by microscopic analysis performed on peripheral nerve sensory axons: abundant vacuolated and swollen mitochondria were observed in the rats treated with paclitaxel to induce pain (Canta et al., 2015; Dai et al., 2020). The increase of mtDNA is associated with pain-like behavior (Trecarichi et al., 2022). Additionally, it has evidenced that mtDNA released and activated the cGAS-STING pathway in low back pain model (Ning et al., 2020). Altogether, this evidence above suggests that mtDNA or nuclear DNA released may activate the cGAS-STING pathway in the process of chronic pain.

### Other PRRs, such as TLRs, may be involved in the activation of cGAS-STING of chronic pain

Several lines of evidence have shown that Toll-like receptors (TLRs) signaling enables the cGAS-STING pathway to induce a strong type 1 interferon response during HIV-1 infection (Siddiqui and Yamashita, 2021). Additionally, TLR4 has been considered essential for activating the cGAS-STING pathway in macrophages stimulated by LPS (Ning et al., 2020), although current studies have suggested that after nerve injury, TLRs are involved in Wallerian degeneration and the generation of neuropathic pain (Thakur et al., 2017). Particularly, TLR3 promotes neuropathic pain by regulating autophagy in rats with L5 spinal nerve ligation model (Chen and Lu, 2017), and knockdown of TLR3 in bone malignancy pain model mice can impair pain thoroughly (Zhang et al., 2020). Furthermore, TLR3 has also been proven to trigger type 1 interferon responses *via* Toll/IL-1 receptor domain containing adaptor inducing IFN-β (TRIF) signaling to regulate pain and itch (Szöllosi et al., 2019). It is suggested that TLRs may be involved in activating the cGAS-STING pathway in chronic pain, although it remains to be examined.

## The main downstream signaling pathways of cGAS-STING in chronic pain

### Type 1 interferons

The cGAS-STING pathway, which controls immunity to cytosolic DNA, is a critical driver of aberrant type 1 interferon responses (Domizio et al., 2022). Furthermore, the latest research on COVID-19 has revealed that type 1 interferon response, which is activated by cGAS-STING pathway, induces microangiopathic changes and produces neuropathic pain in young patients (Papa et al., 2021). Simultaneously, according to a study of hepatitis C virus patients, IFN-α therapy causes significant somatic pain and promotes major depressive disorder as early as the second week of treatment (Lin et al., 2020), although early reports have demonstrated that IFN-α and IFN-β, two major family members of type 1 interferons, exert an antinociceptive action (Menzies et al., 1992). In addition, STING regulates the type 1 interferon signaling in dorsal root ganglion sensory neurons, which has been disclosed to control nociception in bone cancer pain models (Donnelly et al., 2021). Consistently, in chronic constriction injury-induced neuropathic pain, protein tyrosine phosphatase receptor type D (PTPRD) activates STING-type 1 interferon pathway in dorsal root ganglion and displays an analgesic effect (Sun et al., 2022). In the light of this, researchers have reviewed that IFN-α and IFN-β may rapidly suppress neuronal activity and synaptic transmission to potent analgesia *via* nongenomic regulation (Tan et al., 2021), while the discrepancy in antinociceptive against pronociceptive effects of type 1 interferons may ascribe to various conditions, and the exact role of STING/type 1 interferons in chronic pain requires further investigation.

### NLRP3

STING predominantly inhabits the endoplasmic reticulum to regulate innate immune signaling processes and recruits NLRP3 to the ER to promote the inflammasome formation in the HSV-1 infection (Wang et al., 2020). Lipopolysaccharide (LPS) activates STING and afterward upregulates NLRP3 expression in acute lung injury (Ning et al., 2020). Simultaneously, an increasing body of evidence verified that NLRP3 inflammasome controls the processing of proinflammatory cytokine interleukin 1β (IL-1β) and is implicated in chronic pain (Pan et al., 2018; Chen et al., 2021). In another study of lower back pain, NLRP3 activation in nucleus pulposus cells was associated with cGAS and STING and participated in chronic pain (Tian et al., 2020). The inhibitor of NLRP3 inflammasome can effectively modify nitroglycerin-induced mechanical hyperalgesia (He et al., 2019). Therefore, NLRP3 has become an emerging therapeutic target for chronic pain, and it shows promise that STING may regulate NLRP3 to participate in chronic pain.

### NF-κB

Nuclear factor-kappa B (NF-κB) is an important nuclear transcription factor in almost all cell types and participates in numerous biological processes, including inflammation, cell differentiation, immunity, cell growth and apoptosis, and tumorigenesis (Ghosh and Dass, 2016). STING activation can trigger nuclear factor κB (NF-κB) signaling to mediate immune defense against tumors and viral infections (Yum et al., 2021). Simultaneously, STING antagonist H-151 has been shown that suppresses STING/NF-κB-mediated inflammation to ameliorate psoriasis (Pan et al., 2021). Notably, the latest research in neuropathic pain has indicated that STING may activate the NF-κB and release the proinflammatory cytokines IL-6 in the spinal cord to aggravate chronic pain. However, injecting STING antagonist C-176 can provide an analgesic effect and reverse the increased expression of NF-κB (Sun et al., 2021). Additionally, NF-κB is a transcription factor for various cytokines, including CX3CL1, IL-1β, IL-6, and TNF-α, to induce peripheral and central sensitization in chronic pain (Sun et al., 2012; Liu et al., 2018; Xiang et al., 2019; Li Y. et al., 2020; Xu et al., 2021). Undoubtedly, NF-κB activation may contribute to chronic pain; therefore, as an important mediator of NF-κB, STING is supposed to play a significant role in chronic pain.

## The potential mechanism of the cGAS-STING pathway in chronic pain

The extrinsic dsDNA (virus and bacteria), intrinsic dsDNA (nDNA and mtDNA), and other pathogen recognition receptors (PRRs) such as Toll-like receptors (TLRs) may contribute to chronic pain by activating the cGAS-STING pathway. Meanwhile, the downstream proteins of the cGAS-STING pathway, such as type 1 interferons, NLRP3, and NF-κB, have been established that participate in chronic pain. Furthermore, cluster analysis displayed that cGAS-STING activation could affect inflammation, immunity, apoptosis, autophagy, and other signaling pathways (Wu et al., 2020); these cellular processes may involve chronic pain. Based on the preceding evidence, we hypothesized that the cGAS-STING pathway might significantly affect chronic pain.

### cGAS-STING may induce inflammation to contribute to chronic pain

Evidence accumulates that STING pathway is involved in inflammation (Chin, 2019); for instance, STING-mediated type 1 interferons regulates the elevation of proinflammatory cytokine profile with increased TNF-α, IL-6, and IL-1β in microglia of traumatic brain injury (Abdullah et al., 2018). Moreover, these studies have revealed that the cGAS-STING pathway was activated, and the inflammatory cytokines IL-6 and CXCL10 were released in mitochondrial dysfunction (Zhou et al., 2021). Additionally, in cerebral ischemic stroke, cGAS knockdown promotes microglial M2 polarization to alleviate inflammation (Jiang et al., 2021). Emerging evidence suggested that inflammation plays an essential role in chronic pain (Ji et al., 2016, 2018): The upregulation of inflammatory mediators (TNF-α, IL-6, and IL-1β) in the spinal cord has been evidenced that might be involved in the process of bone cancer pain. Inhibition of endoplasmic reticulum stress may decrease the inflammatory mediators and show an antinociception effect (Wei et al., 2016; Mao et al., 2020). Particularly, in inflammatory pain, magnetic resonance imaging (MRI) and histopathology have demonstrated that inflammation caused by cGAS, STING, and NLRP3 is associated with intervertebral disc degeneration in low back pain (Zhang et al., 2022). Moreover, STING can also participate in the inflammatory reaction process of neuropathic pain. In spinal cord injury mice, STING knockout may alleviate inflammatory response (Wang et al., 2019); in spared nerve injury rats, STING inhibitor C-176 administration provides an antinociceptive effect that is reversed by injecting recombinant IL-6 (Sun et al., 2021), which indicates that STING is an essential downstream factor for chronic pain by regulating IL-6 expression. Accordingly, it would not surprise that activation of the cGAS-STING pathway may induce inflammation to mediate inflammatory and neuropathic pain.

### cGAS-STING may mediate immunity to participate in chronic pain

The cGAS-STING pathway has a significant role in the immune, and emerging studies provide solid evidence that cGAS-STING is mainly attributed to antigen-presenting cell (APC)-mediated activation of CD8^+^ T cells (Flood et al., 2019) and spontaneous CD8^+^ T cell priming against tumors was defective in mice lacking STING (Woo et al., 2014). Meanwhile, cGAS-STING-mediated DNA sensing has been reported that maintains CD8^+^ T cell stemness and promotes anti-tumor T cell therapy (Li W. et al., 2020). Although the clinical research disclosed that STING agonist ADU-S100 might induce CD8^+^ T cell immune response at a low dose, when administered at a high dose, the CD8^+^ T cell death, and compromised anti-tumor immunity (Sivick et al., 2018), it is no doubt that STING mediates CD8^+^ T cells.

A variety of inflammatory pain processes are believed to be mediated by T cells, including rheumatoid arthritis, intestinal inflammation, etc. (Basso et al., 2015; Swain et al., 2022). Recent studies have found that T cells are also involved in neuropathic pain: CD8^+^ T cells and endogenous IL-10 are required to resolve chemotherapy-induced neuropathic pain (Krukowski et al., 2016); educating CD8^+^ T cells can prevent and resolve chemotherapy-induced peripheral neuropathy in mice (Laumet et al., 2019; Singh et al., 2022). Intriguingly, in the bone cancer pain model which may involve both inflammatory pain and neuropathic pain, systemic administration of STING activator DMXAA attenuated the measures of spontaneous and ongoing pain; the analgesic effect may attribute to the increasing proportion of CD8^+^ T cells in the bone marrow tumor microenvironment (Donnelly et al., 2021). Therefore, these findings suggest that the cGAS-STING pathway may involve chronic pain by mediating the immunity of CD8^+^ T cells.

### cGAS-STING may induce autophagy to contribute to chronic pain

Autophagy is a lysosomal degradation pathway that maintains tissue homeostasis by recycling damaged and aged cellular components, which plays a significant role in developing the nervous system, neuronal function, and survival (Liu X. et al., 2019). Emerging studies showed that innate immunity-related proteins control the progress of autophagy (Wild et al., 2011). Extracellular M. tuberculosis DNA induces autophagy by activating the cGAS-STING pathway, suggesting a link between STING and autophagy (Watson et al., 2012). Additionally, injecting the activator of IRF3, the downstream protein of the cGAS-STING pathway, can inhibit the fusion of the autophagosome with the lysosome to reduce autophagy flux (67), indicating that the activation of the STING pathway may impair the autophagy. In neuropathic pain, autophagy flux is impaired and mainly exists in astrocytes during the maintenance of the study by Li et al. (2021) and Liao et al. (2022); no matter the stage of neuropathic pain induction or maintenance, upregulation of autophagy can suppresses pain behavior (Li et al., 2021). Researchers suggest that upregulated autophagic activities may facilitate myelin clearance, promote nerve regeneration, and reduce pain. Furthermore, the cGAS-STING pathway has been reported to directly interact with microtubule-associated protein light chain 3 (LC3) and subsequent autophagy to regulate the innate immune responses (Liu D. et al., 2019). The expression of LC3 is upregulated in GABAergic interneurons of rat spinal dorsal horn after SNL, suggesting that autophagic disruption following SNL might be involved in the induction and maintenance of neuropathic pain (Liu X. et al., 2019). Intrathecal injection of autophagy activator exerts an analgesic effect on neuropathic pain by activating LC3 in astrocytes (Yuan and Fei, 2021). It can be suggested that STING may interact with LC3 to aggravate chronic pain further. The role of autophagy in inflammatory pain has been studied relatively less, while a study by Liu found that CFA-induced inflammatory pain can be alleviated by restoring autophagic flux in the spinal cord (Liu et al., 2022). Autophagy may mediate both inflammatory and neuropathic pain, although there is no solid evidence to confirm this hypothesis, it is still appropriate to predict that STING may mediate autophagy flux and contribute to chronic pain.

### cGAS-STING may induce apoptosis to contribute to chronic pain

Apoptosis is a form of programmed cell death and shrinkage when cells encounter stress or damage (Liao et al., 2022). There is evidence for this occurring that STING-dependent apoptosis directly through the interaction of IRF3 and BCL2-associated x, apoptosis regulator (Bax) (Sze et al., 2013), and in microglia and other immune cells, HSV-1 at a high viral load can trigger cGAS/STING-dependent apoptosis (Reinert et al., 2021). Additionally, STING-IRF3 contributes to lipopolysaccharide-induced cardiac apoptosis *via* activating NLRP3 (Li et al., 2019). Consequently, cGAS-STING pathway is essential for apoptosis regulation. Furthermore, apoptotic activity in the injured nerve, dorsal root ganglia, and spinal cord has changed during neuropathic pain formation (Liao et al., 2022). The number of cleaved caspase-3 in the spinal cord increased on days 8 and 14 after nerve injury (Fu et al., 2017). Meanwhile, increased spinal cell apoptosis may probably induce inflammatory pain (Baniasadi et al., 2020). Although there is no clear evidence demonstrating that inhibiting apoptotic activities could attenuate pain behavior, daily hyperbaric oxygen therapy has been proved to suppress pain behavior and reduce apoptotic activities of GABAergic neurons in the spinal dorsal horn of rats (Fu et al., 2017). The apoptosis level was increased in osteoarthritic, which develops slowly and worsens over time, accompanied by chronic pain (Aigner et al., 2001); recently, it has been evidenced that STING might induce apoptosis and senescence in chondrocytes *via* NF-κB pathway in osteoarthritic mice; STING knockdown may reduce the level of chondrocyte apoptosis and ameliorate the symptom (Guo et al., 2021). Simultaneously, epigallocatechin gallate also has been found that reduces the apoptotic rate *via* inhibiting the cGAS-STING pathway activation in nucleus pulposus cells of low back pain models (Tian et al., 2020). The process of apoptosis may contribute to the pathology of inflammatory or neuropathic pain. In view of cGAS-STING that can promote apoptosis in various diseases, it is proposed that cGAS-STING may induce apoptosis and promote chronic pain.

## Concluding remarks and future perspectives

The cGAS-STING axis is initially recognized as the an essential mechanism for the host to defeat bacteria and virus invasions (Ding et al., 2020), as the crucial role in the neuro-immune, activation of immunostimulatory pathways is required for normal functioning, but the persistent engagement of these responses can prove detrimental.

It was found in the recent studies that the cGAS-STING may be an important mediator of chronic pain, and this review summarized and discussed the potential relationship. First, pathogens and damaged cells will release exogenous and endogenous DNA, which may activate the cGAS-STING pathway and then contribute to chronic pain. Additionally, TLRs were activated in chronic pain, which may be in concert with the cGAS-STING pathway. As such, it is unsurprising that the cGAS-STING pathway may be activated in chronic pain; however, the different types and mechanisms of DNA and TLRs should be further explored. Moreover, the activated STING further exerted its effect by promoting the expressions of type 1 interferons, NF-κB, and NLRP3. Intriguingly, the downstream has been shown to play a significant role in chronic pain. It can be conceived that the cGAS-STING pathway may mediate chronic pain by activating these downstream; however, more mechanisms are required for further research. Besides, STING may influence inflammation, immunity (such as CD8^+^ T cells), autophagy, and apoptosis, which has been proved to be the essential cause of chronic pain. Particularly, these cellular processes may influence each other in chronic pain; for example, LC3-associated phagocytosis can assist remove apoptotic cells by macrophages and inhibit proinflammatory processes (Eid and Ito, 2022). The relevance of these branches of cGAS/STING pathway for their potential role in chronic pain is currently not well-understood. Further research may be required to provide a more comprehensive picture of STING in chronic pain and pain-related cellular signals.

What role cGAS-STING plays in chronic pain is still controversial. Agonists and inhibitors targeting cGAS-STING have been developed with potentials for the treatment of auto-inflammation, virus infection, and cancers (Ding et al., 2020). In chronic-refractory pain, STING-related adjuvant therapy appears to have translational potential, and the latest research has reported that a small molecule drug 7-BIA could alleviate neuropathic pain by upregulating STING and IFN-α in the DRG (Sun et al., 2022). There is a possibility of developing new drugs that target the STING signal pathway to treat refractory cancer-induced bone pain or neuropathic pain in patients to avoid systemic effects.

STING enhances the production and secretion of type 1 interferons (IFN-α and IFN-ß). As we know, IFN-α and IFN-ß have already been successfully used for adjuvant therapy in malignancy, infection, and autoimmune disease. As well as trials of IFN-α, STING agonists more recently used for adjuvant anti-malignancy treatment were also tested (Lv et al., 2020; Verdegaal et al., 2020; Meric-Bernstam et al., 2022). It is therefore possible to determine whether low-dose IFN-α and STING agonists reduced or induced pain by examining existing datasets and patients previously treated with this drug.

Additionally, STING's effect on nociceptors, which affect immune cells (Donnelly et al., 2021), might be an interesting target in basic science. If immune-mediated attenuation of STING's analgesic function occurs, then the analgesic effects of STING could be further enhanced by blocking (currently unknown) immune-mediated pro-analgesic effects. What is the mechanism by which immune cells modulate STING's analgesic effects? How the new STING analgesic signaling plays out in young/old subjects, and whether male/female sex differences exist? There are still questions to be answered.

## Author contributions

JW, XL, and XZ searched the literature and participated in manuscript preparation. WW, JW, and XY made the critical revision of the manuscript. All authors have read and approved the final manuscript.

## Funding

This work was supported by the National Natural Science Foundation of China (grant No. 82071233), Shanghai Sailing Program (grant No. 21YF1442900), and Nurture projects for basic research of Shanghai Chest Hospital (grant Nos. 2019YNJCM08, 2020YNJCQ13).

## Conflict of interest

The authors declare that the research was conducted in the absence of any commercial or financial relationships that could be construed as a potential conflict of interest.

## Publisher's note

All claims expressed in this article are solely those of the authors and do not necessarily represent those of their affiliated organizations, or those of the publisher, the editors and the reviewers. Any product that may be evaluated in this article, or claim that may be made by its manufacturer, is not guaranteed or endorsed by the publisher.

## References

[B1] Abboud H. Abboud F. Z. Kharbouch H. Arkha Y. El Abbadi N. El Ouahabi A. (2020). COVID-19 and SARS-CoV-2 infection: pathophysiology and clinical effects on the nervous system. World Neurosurg. 140, 49–53. 10.1016/j.wneu.2020.05.193 32474093 PMC7255736

[B2] Abdullah A. Zhang M. Frugier T. Bedoui S. Taylor J. M. Crack P. J. (2018). STING-mediated type-I interferons contribute to the neuroinflammatory process and detrimental effects following traumatic brain injury. J. Neuroinflamm. 15, 323. 10.1186/s12974-018-1354-7 30463579 PMC6247615

[B3] Ablasser A. Schmid-Burgk J. L. Hemmerling I. Horvath G. L. Schmidt T. Latz E.. (2013). Cell intrinsic immunity spreads to bystander cells via the intercellular transfer of cGAMP. Nature 503, 530–534. 10.1038/nature12640 24077100 PMC4142317

[B4] Ablasser A. Chen Z. J. (2019). cGAS in action: expanding roles in immunity and inflammation. Science 363, eaat8657. 10.1126/science.aat8657 30846571

[B5] Aigner T. Hemmel M. Neureiter D. Gebhard P. M. Zeiler G. Kirchner T.. (2001). Apoptotic cell death is not a widespread phenomenon in normal aging and osteoarthritis human articular knee cartilage: a study of proliferation, programmed cell death (apoptosis), and viability of chondrocytes in normal and osteoarthritic human knee cartilage. Arthrit. Rheum. 44, 1304–1312. 10.1002/1529-0131(200106)44:6<1304::AID-ART222>3.0.CO 11407689

[B6] Baniasadi M. Manaheji H. Maghsoudi N. Danyali S. Zakeri Z. Maghsoudi A.. (2020). Microglial-induced apoptosis is potentially responsible for hyperalgesia variations during CFA-induced inflammation. Inflammopharmacology 28, 475–485. 10.1007/s10787-019-00623-3 31388881

[B7] Barragán-Iglesias P. Franco-Enzástiga Ú. Jeevakumar V. Shiers S. Wangzhou A. Granados-Soto V.. (2020). Type I interferons act directly on nociceptors to produce pain sensitization: implications for viral infection-induced pain. J. Neurosci. 40, 3517–3532. 10.1523/JNEUROSCI.3055-19.2020 32245829 PMC7189756

[B8] Basso L. Bourreille A. Dietrich G. (2015). Intestinal inflammation and pain management. Curr. Opin. Pharmacol. 25, 50–55. 10.1016/j.coph.2015.11.004 26629597

[B9] Bhattacharya S. Srinivasan K. Abdisalaam S. Su F. Raj P. Dozmorov I.. (2017). RAD51 interconnects between DNA replication, DNA repair and immunity. Nucleic Acids Res. 45, 4590–4605. 10.1093/nar/gkx126 28334891 PMC5416901

[B10] Brush D. E. (2012). Complications of long-term opioid therapy for management of chronic pain: the paradox of opioid-induced hyperalgesia. J. Med. Toxicol. 8, 387–392. 10.1007/s13181-012-0260-0 22983894 PMC3550256

[B11] Burdette D. L. Monroe K. M. Sotelo-Troha K. Iwig J. S. Eckert B. Hyodo M.. (2011). STING is a direct innate immune sensor of cyclic di-GMP. Nature 478, 515–518. 10.1038/nature10429 21947006 PMC3203314

[B12] Canta A. Pozzi E. Carozzi V. A. (2015). Mitochondrial dysfunction in chemotherapy-induced peripheral neuropathy (CIPN). Toxics 3, 198–223. 10.3390/toxics3020198 29056658 PMC5634687

[B13] Chen R. Yin C. Fang J. Liu B. (2021). The NLRP3 inflammasome: an emerging therapeutic target for chronic pain. J. Neuroinflamm. 18, 84. 10.1186/s12974-021-02131-0 33785039 PMC8008529

[B14] Chen W. Lu Z. (2017). Upregulated TLR3 promotes neuropathic pain by regulating autophagy in rat with L5 spinal nerve ligation model. Neurochem. Res. 42, 634–643. 10.1007/s11064-016-2119-2 28000161

[B15] Chin A. C. (2019). Neuroinflammation and the cGAS-STING pathway. J. Neurophysiol. 121, 1087–1091. 10.1152/jn.00848.2018 30673358

[B16] Couillin I. Riteau N. (2021). STING signaling and sterile inflammation. Front. Immunol. 12, 753789. 10.3389/fimmu.2021.753789 34659260 PMC8517477

[B17] Dai C. Q. Guo Y. Chu X. Y. (2020). Neuropathic pain: the dysfunction of Drp1, mitochondria, and ROS homeostasis. Neurotox. Res. 38, 553–563. 10.1007/s12640-020-00257-2 32696439

[B18] Descalzi G. Ikegami D. Ushijima T. Nestler E. J. Zachariou V. Narita M. (2015). Epigenetic mechanisms of chronic pain. Trends Neurosci. 38, 237–246. 10.1016/j.tins.2015.02.001 25765319 PMC4459752

[B19] Diamond J. M. Vanpouille-Box C. Spada S. Rudqvist N. P. Chapman J. R. Ueberheide B. M.. (2018). Exosomes shuttle TREX1-sensitive IFN-stimulatory dsDNA from irradiated cancer cells to DCs. Cancer Immunol. Res. 6, 910–920. 10.1158/2326-6066.CIR-17-0581 29907693 PMC6072562

[B20] Ding C. Song Z. Shen A. Chen T. Zhang A. (2020). Small molecules targeting the innate immune cGAS–STING–TBK1 signaling pathway. Acta Pharm. Sin. B 10, 2272–2298. 10.1016/j.apsb.2020.03.001 33354501 PMC7745059

[B21] Domizio J. D. Gulen M. F. Saidoune F. Thacker V. V. Yatim A. Sharma K.. (2022). The cGAS-STING pathway drives type I IFN immunopathology in COVID-19. Nature 603, 145–151. 10.1038/s41586-022-04421-w 35045565 PMC8891013

[B22] Donnelly C. R. Jiang C. Andriessen A. S. Wang K. Wang Z. Ding H.. (2021). STING controls nociception via type I interferon signalling in sensory neurons. Nature 591, 275–280. 10.1038/s41586-020-03151-1 33442058 PMC7977781

[B23] Eid N. Ito Y. (2022). Oxoglaucine alleviates osteoarthritis by activation of autophagy via blockade of Ca(2+) influx and TRPV5/calmodulin/CAMK-II pathway. Br. J. Pharmacol. 179, 1282–1283. 10.1111/bph.15706 34734425

[B24] Flood B. A. Higgs E. F. Li S. Luke J. J. Gajewski T. F. (2019). STING pathway agonism as a cancer therapeutic. Immunol. Rev. 290, 24–38. 10.1111/imr.12765 31355488 PMC6814203

[B25] Fu H. Li F. Thomas S. Yang Z. (2017). Hyperbaric oxygenation alleviates chronic constriction injury (CCI)-induced neuropathic pain and inhibits GABAergic neuron apoptosis in the spinal cord. Scand J. Pain 17, 330–338. 10.1016/j.sjpain.2017.08.014 28927648

[B26] Ghosh S. Dass J. F. P. (2016). Study of pathway cross-talk interactions with NF-κB leading to its activation via ubiquitination or phosphorylation: a brief review. Gene 584, 97–109. 10.1016/j.gene.2016.03.008 26968890

[B27] Guo Q. Chen X. Chen J. Zheng G. Xie C. Wu H.. (2021). STING promotes senescence, apoptosis, and extracellular matrix degradation in osteoarthritis via the NF-κB signaling pathway. Cell Death Dis. 12, 13. 10.1038/s41419-020-03341-9 33414452 PMC7791051

[B28] He W. Long T. Pan Q. Zhang S. Zhang Y. Zhang D.. (2019). Microglial NLRP3 inflammasome activation mediates IL-1β release and contributes to central sensitization in a recurrent nitroglycerin-induced migraine model. J. Neuroinflamm. 16, 78. 10.1186/s12974-019-1459-7 30971286 PMC6456991

[B29] Ishikawa H. Barber G. N. (2008). STING is an endoplasmic reticulum adaptor that facilitates innate immune signalling. Nature 455, 674–678. 10.1038/nature07317 18724357 PMC2804933

[B30] Ji R. R. Chamessian A. Zhang Y. Q. (2016). Pain regulation by non-neuronal cells and inflammation. Science 354, 572–577. 10.1126/science.aaf8924 27811267 PMC5488328

[B31] Ji R. R. Nackley A. Huh Y. Terrando N. Maixner W. (2018). Neuroinflammation and central sensitization in chronic and widespread pain. Anesthesiology 129, 23. 10.1097/ALN.0000000000002130 29462012 PMC6051899

[B32] Jiang G. L. Yang X. L. Zhou H. J. Long J. Liu B. Zhang L. M.. (2021). cGAS knockdown promotes microglial M2 polarization to alleviate neuroinflammation by inhibiting cGAS-STING signaling pathway in cerebral ischemic stroke. Brain Res. Bull. 171, 183–195. 10.1016/j.brainresbull.2021.03.010 33745949

[B33] Kato J. Agalave N. M. Svensson C. I. (2016). Pattern recognition receptors in chronic pain: mechanisms and therapeutic implications. Eur. J. Pharmacol. 788, 261–273. 10.1016/j.ejphar.2016.06.039 27343378

[B34] Krukowski K. Eijkelkamp N. Laumet G. Hack C. E. Li Y. Dougherty P. M.. (2016). CD8+ T cells and endogenous IL-10 are required for resolution of chemotherapy-induced neuropathic pain. J. Neurosci. 36, 11074–11083. 10.1523/JNEUROSCI.3708-15.2016 27798187 PMC5098842

[B35] Laumet G. Edralin J. D. Dantzer R. Heijnen C. J. Kavelaars A. (2019). Cisplatin educates CD8+ T cells to prevent and resolve chemotherapy-induced peripheral neuropathy in mice. Pain 160, 1459–1468. 10.1097/j.pain.0000000000001512 30720585 PMC6527475

[B36] Li J. Tian M. Hua T. Wang H. Yang M. Li W.. (2021). Combination of autophagy and NFE2L2/NRF2 activation as a treatment approach for neuropathic pain. Autophagy 17, 4062–4082. 10.1080/15548627.2021.1900498 33834930 PMC8726676

[B37] Li N. Zhou H. Wu H. Wu Q. Duan M. Deng W.. (2019). STING-IRF3 contributes to lipopolysaccharide-induced cardiac dysfunction, inflammation, apoptosis and pyroptosis by activating NLRP3. Redox Biol. 24, 101215. 10.1016/j.redox.2019.101215 31121492 PMC6529775

[B38] Li W. Lu L. Lu J. Wang X. Yang C. Jin J.. (2020). cGAS-STING-mediated DNA sensing maintains CD8(+) T cell stemness and promotes antitumor T cell therapy. Sci. Transl. Med. 12, eaay9013. 10.1126/scitranslmed.aay9013 32581136

[B39] Li Y. Yang Y. Guo J. Guo X. Feng Z. Zhao X. (2020). Spinal NF-kB upregulation contributes to hyperalgesia in a rat model of advanced osteoarthritis. Mol. Pain 16, 1744806920905691. 10.1177/1744806920905691 31971058 PMC7040927

[B40] Liao M. F. Lu K. T. Hsu J. L. Lee C. H. Cheng M. Y. Ro L. S. (2022). The role of autophagy and apoptosis in neuropathic pain formation. Int. J. Mol. Sci. 23, 2685. 10.3390/ijms23052685 35269822 PMC8910267

[B41] Lin C. Y. Guu T. W. Lai H. C. Peng C. Y. Chiang J. Y. Chen H. T.. (2020). Somatic pain associated with initiation of interferon-alpha (IFN-α) plus ribavirin (RBV) therapy in chronic HCV patients: a prospective study. Brain Behav. Immun. Health 2, 100035. 10.1016/j.bbih.2019.100035 34589826 PMC8474510

[B42] Liu C. Zhang F. Liu H. Wei F. (2018). NF-kB mediated CX3CL1 activation in the dorsal root ganglion contributes to the maintenance of neuropathic pain induced in adult male Sprague Dawley rats1. Acta Cir. Bras. 33, 619–628. 10.1590/s0102-865020180070000007 30110063

[B43] Liu C. Zheng X. Liu L. Hu Y. Zhu Q. Zhang J.. (2022). Caloric restriction alleviates CFA-induced inflammatory pain via elevating β-hydroxybutyric acid expression and restoring autophagic flux in the spinal cord. Front. Neurosci. 16, 828278. 10.3389/fnins.2022.828278 35573301 PMC9096081

[B44] Liu D. Wu H. Wang C. Li Y. Tian H. Siraj S.. (2019). STING directly activates autophagy to tune the innate immune response. Cell Death Differ. 26, 1735–1749. 10.1038/s41418-018-0251-z 30568238 PMC6748081

[B45] Liu X. Zhu M. Ju Y. Li A. Sun X. (2019). Autophagy dysfunction in neuropathic pain. Neuropeptides 75, 41–48. 10.1016/j.npep.2019.03.005 30910234

[B46] Lv M. Chen M. Zhang R. Zhang W. Wang C. Zhang Y.. (2020). Manganese is critical for antitumor immune responses via cGAS-STING and improves the efficacy of clinical immunotherapy. Cell Res. 30, 966–979. 10.1038/s41422-020-00395-4 32839553 PMC7785004

[B47] Mao Y. Wang C. Tian X. Huang Y. Zhang Y. Wu H.. (2020). Endoplasmic reticulum stress contributes to nociception via neuroinflammation in a murine bone cancer pain model. Anesthesiology 132, 357–372. 10.1097/ALN.0000000000003078 31939851

[B48] Menzies R. A. Patel R. Hall N. R. O'Grady M. P. Rier S. E. (1992). Human recombinant interferon alpha inhibits naloxone binding to rat brain membranes. Life Sci. 50, Pl227–232. 10.1016/0024-3205(92)90555-4 1317938

[B49] Meric-Bernstam F. Sweis R. F. Hodi F. S. Messersmith W. A. Andtbacka R. H. I. Ingham M.. (2022). Phase I dose-escalation trial of MIW815 (ADU-S100), an intratumoral STING agonist, in patients with advanced/metastatic solid tumors or lymphomas. Clin. Cancer Res. 28, 677–688. 10.1158/1078-0432.CCR-21-1963 34716197

[B50] Ning L. Wei W. Wenyang J. Rui X. Qing G. (2020). Cytosolic DNA-STING-NLRP3 axis is involved in murine acute lung injury induced by lipopolysaccharide. Clin. Transl. Med. 10, e228. 10.1002/ctm2.228 33252860 PMC7668192

[B51] Pan Y. You Y. Sun L. Sui Q. Liu L. Yuan H.. (2021). The STING antagonist H-151 ameliorates psoriasis via suppression of STING/NF-κB-mediated inflammation. Br. J. Pharmacol. 178, 4907–4922. 10.1111/bph.15673 34460100

[B52] Pan Z. Shan Q. Gu P. Wang X. M. Tai L. W. Sun M.. (2018). miRNA-23a/CXCR4 regulates neuropathic pain via directly targeting TXNIP/NLRP3 inflammasome axis. J. Neuroinflamm. 15, 29. 10.1186/s12974-018-1073-0 29386025 PMC5791181

[B53] Papa A. Salzano A. M. Di Dato M. T. Lo Bianco G. Tedesco M. Salzano A.. (2021). COVID-19 related acro-ischemic neuropathic-like painful lesions in pediatric patients: a case series. Anesth. Pain Med. 11, e113760. 10.5812/aapm.113760 34336629 PMC8314085

[B54] Reinert L. S. Rashidi A. S. Tran D. N. Katzilieris-Petras G. Hvidt A. K. Gohr M.. (2021). Brain immune cells undergo cGAS/STING-dependent apoptosis during herpes simplex virus type 1 infection to limit type I IFN production. J. Clin. Invest. 131, e136824. 10.1172/JCI136824 32990676 PMC7773356

[B55] Siddiqui M. A. Yamashita M. (2021). Toll-like receptor (TLR) signaling enables cyclic GMP-AMP synthase (cGAS) sensing of HIV-1 infection in macrophages. MBio 12, e0281721. 10.1128/mBio.02817-21 34844429 PMC8630538

[B56] Singh S. K. Krukowski K. Laumet G. O. Weis D. Alexander J. F. Heijnen C. J.. (2022). CD8+ T cell-derived IL-13 increases macrophage IL-10 to resolve neuropathic pain. JCI Insight 7, e154194. 10.1172/jci.insight.154194 35260535 PMC8983134

[B57] Sivick K. E. Desbien A. L. Glickman L. H. Reiner G. L. Corrales L. Surh N. H.. (2018). Magnitude of therapeutic STING activation determines CD8(+) T cell-mediated anti-tumor immunity. Cell Rep. 25, 3074–3085.e3075. 10.1016/j.celrep.2018.11.047 30540940

[B58] Sun C. Wu G. Zhang Z. Cao R. Cui S. (2022). Protein tyrosine phosphatase receptor type D regulates neuropathic pain after nerve injury via the STING-IFN-I pathway. Front. Mol. Neurosci. 15, 859166. 10.3389/fnmol.2022.859166 35493326 PMC9047945

[B59] Sun J. Zhou Y. Q. Xu B. Y. Li J. Y. Zhang L. Q. Li D. Y.. (2021). STING/NF-κB/IL-6-mediated inflammation in microglia contributes to spared nerve injury (SNI)-induced pain initiation. J. Neuroimmune Pharmacol. 10.1007/s11481-021-10031-6 34727296

[B60] Sun T. Luo J. Jia M. Li H. Li K. Fu Z. (2012). Small interfering RNA-mediated knockdown of NF-κBp65 attenuates neuropathic pain following peripheral nerve injury in rats. Eur. J. Pharmacol. 682, 79–85. 10.1016/j.ejphar.2012.02.017 22381070

[B61] Swain N. Tripathy A. Padhan P. Raghav S. K. Gupta B. (2022). Toll-like receptor-7 activation in CD8+ T cells modulates inflammatory mediators in patients with rheumatoid arthritis. Rheumatol. Int. 42, 1235–1245. 10.1007/s00296-021-05050-8 35142867

[B62] Sze A. Belgnaoui S. M. Olagnier D. Lin R. Hiscott J. van Grevenynghe J. (2013). Host restriction factor SAMHD1 limits human T cell leukemia virus type 1 infection of monocytes via STING-mediated apoptosis. Cell Host Microbe 14, 422–434. 10.1016/j.chom.2013.09.009 24139400

[B63] Szöllosi A. G. McDonald I. Szabó I. L. Meng J. van den Bogaard E. Steinhoff M. (2019). TLR3 in chronic human itch: a keratinocyte-associated mechanism of peripheral itch sensitization. J. Invest. Dermatol. 139, 2393–2396.e2396. 10.1016/j.jid.2019.04.018 31129058

[B64] Tan P. H. Ji J. Yeh C. C. Ji R. R. (2021). Interferons in pain and infections: emerging roles in neuro-immune and neuro-glial interactions. Front. Immunol. 12, 783725. 10.3389/fimmu.2021.783725 34804074 PMC8602180

[B65] Tarnanen S. Neva M. H. Dekker J. Häkkinen K. Vihtonen K. Pekkanen L.. (2012). Randomized controlled trial of postoperative exercise rehabilitation program after lumbar spine fusion: study protocol. BMC Musculoskelet. Disord. 13, 123. 10.1186/1471-2474-13-123 22817607 PMC3436790

[B66] Thakur K. K. Saini J. Mahajan K. Singh D. Jayswal D. P. Mishra S.. (2017). Therapeutic implications of toll-like receptors in peripheral neuropathic pain. Pharmacol. Res. 115, 224–232. 10.1016/j.phrs.2016.11.019 27894923

[B67] Thompson M. R. Kaminski J. J. Kurt-Jones E. A. Fitzgerald K. A. (2011). Pattern recognition receptors and the innate immune response to viral infection. Viruses 3, 920–940. 10.3390/v3060920 21994762 PMC3186011

[B68] Tian Y. Bao Z. Ji Y. Mei X. Yang H. (2020). Epigallocatechin-3-gallate protects H(2)O(2)-induced nucleus pulposus cell apoptosis and inflammation by inhibiting cGAS/Sting/NLRP3 activation. Drug Des. Devel. Ther. 14, 2113–2122. 10.2147/DDDT.S251623 32546974 PMC7266312

[B69] Trecarichi A. Duggett N. A. Granat L. Lo S. Malik A. N. Zuliani-Álvarez L.. (2022). Preclinical evidence for mitochondrial DNA as a potential blood biomarker for chemotherapy-induced peripheral neuropathy. PLoS ONE 17, e0262544. 10.1371/journal.pone.0262544 35015774 PMC8752024

[B70] Verdegaal E. van der Kooij M. K. Visser M. van der Minne C. de Bruin L. Meij P.. (2020). Low-dose interferon-alpha preconditioning and adoptive cell therapy in patients with metastatic melanoma refractory to standard (immune) therapies: a phase I/II study. J. Immunother. Cancer 8, e000166. 10.1136/jitc-2019-000166 32238469 PMC7174065

[B71] Wan D. Jiang W. Hao J. (2020). Research advances in how the cGAS-STING pathway controls the cellular inflammatory response. Front. Immunol. 11, 615. 10.3389/fimmu.2020.00615 32411126 PMC7198750

[B72] Wang W. Hu D. Wu C. Feng Y. Li A. Liu W.. (2020). STING promotes NLRP3 localization in ER and facilitates NLRP3 deubiquitination to activate the inflammasome upon HSV-1 infection. PLoS Pathog. 16, e1008335. 10.1371/journal.ppat.1008335 32187211 PMC7080238

[B73] Wang Y. Y. Shen D. Zhao L. J. Zeng N. Hu T. H. (2019). Sting is a critical regulator of spinal cord injury by regulating microglial inflammation via interacting with TBK1 in mice. Biochem. Biophys. Res. Commun. 517, 741–748. 10.1016/j.bbrc.2019.07.125 31400857

[B74] Watson R. O. Manzanillo P. S. Cox J. S. (2012). Extracellular *M. tuberculosis* DNA targets bacteria for autophagy by activating the host DNA-sensing pathway. Cell 150, 803–815. 10.1016/j.cell.2012.06.040 22901810 PMC3708656

[B75] Wei T. Guo T.-Z. Li W.-W. Kingery W. S. Clark J. D. (2016). Acute versus chronic phase mechanisms in a rat model of CRPS. J. Neuroinflammation 13, 14. 10.1186/s12974-015-0472-8 26785976 PMC4719337

[B76] Wild P. Farhan H. McEwan D. G. Wagner S. Rogov V. V. Brady N. R.. (2011). Phosphorylation of the autophagy receptor optineurin restricts Salmonella growth. Science 333, 228–233. 10.1126/science.1205405 21617041 PMC3714538

[B77] Woo S. R. Fuertes M. B. Corrales L. Spranger S. Furdyna M. J. Leung M. Y.. (2014). STING-dependent cytosolic DNA sensing mediates innate immune recognition of immunogenic tumors. Immunity 41, 830–842. 10.1016/j.immuni.2014.10.017 25517615 PMC4384884

[B78] Wu J. Dobbs N. Yang K. Yan N. (2020). Interferon-independent activities of mammalian STING mediate antiviral response and tumor immune evasion. Immunity 53, 115–126.e115. 10.1016/j.immuni.2020.06.009 32640258 PMC7365768

[B79] Xiang H. C. Lin L. X. Hu X. F. Zhu H. Li H. P. Zhang R. Y.. (2019). AMPK activation attenuates inflammatory pain through inhibiting NF-κB activation and IL-1β expression. J. Neuroinflamm. 16, 34. 10.1186/s12974-019-1411-x 30755236 PMC6373126

[B80] Xu M. Fei Y. He Q. Fu J. Zhu J. Tao J.. (2021). Electroacupuncture attenuates cancer-induced bone pain via NF-κB/CXCL12 signaling in midbrain periaqueductal gray. ACS Chem. Neurosci. 12, 3323–3334. 10.1021/acschemneuro.1c00224 34460214

[B81] Yuan J. Fei Y. (2021). Lidocaine activates autophagy of astrocytes and ameliorates chronic constriction injury-induced neuropathic pain. J. Biochem. 170, 25–31. 10.1093/jb/mvaa136 33245112

[B82] Yum S. Li M. Fang Y. Chen Z. J. (2021). TBK1 recruitment to STING activates both IRF3 and NF-κB that mediate immune defense against tumors and viral infections. Proc. Natl. Acad. Sci. U. S. A. 118, e2100225. 10.1073/pnas.2100225118 33785602 PMC8040795

[B83] Zhang W. Li G. Luo R. Lei J. Song Y. Wang B.. (2022). Cytosolic escape of mitochondrial DNA triggers cGAS-STING-NLRP3 axis-dependent nucleus pulposus cell pyroptosis. Exp. Mol. Med. 54, 129–142. 10.1038/s12276-022-00729-9 35145201 PMC8894389

[B84] Zhang Z. Zhang X. Zhang Y. Li J. Xing Z. Zhang Y. (2020). Spinal circRNA-9119 suppresses nociception by mediating the miR-26a-TLR3 axis in a bone cancer pain mouse model. J. Mol. Neurosci. 70, 9–18. 10.1007/s12031-019-01378-w 31368062

[B85] Zhou L. Zhang Y. F. Yang F. H. Mao H. Q. Chen Z. Zhang L. (2021). Mitochondrial DNA leakage induces odontoblast inflammation via the cGAS-STING pathway. Cell Commun. Signal. 19, 58. 10.1186/s12964-021-00738-7 34016129 PMC8136190

